# MiR155 modulates vascular calcification by regulating Akt‐FOXO3a signalling and apoptosis in vascular smooth muscle cells

**DOI:** 10.1111/jcmm.16107

**Published:** 2020-11-18

**Authors:** Yong Li, Wei Sun, Fatma Saaoud, Yuzhen Wang, Quanyi Wang, Johnie Hodge, Yvonne Hui, Sophia Yin, Susan M. Lessner, Xiangqing Kong, Daping Fan

**Affiliations:** ^1^ Department of Cell Biology and Anatomy University of South Carolina School of Medicine Columbia SC USA; ^2^ Department of Cardiology and Department of Cardiothoracic Surgery The First Affiliated Hospital of Nanjing Medical University Nanjing China

**Keywords:** Akt, apoptosis, FOXO3a, microRNA‐155, vascular calcification, vascular smooth muscle cell

## Abstract

microRNA‐155 (miR155) is pro‐atherogenic; however, its role in vascular calcification is unknown. In this study, we aim to examine whether miR155 regulates vascular calcification and to understand the underlying mechanism. Quantitative real‐time PCR showed that miR155 is highly expressed in human calcific carotid tissue and positively correlated with the expression of osteogenic genes. Wound‐healing assay and TUNEL staining showed deletion of miR155 inhibited vascular smooth muscle cell (VSMC) migration and apoptosis. miR155 deficiency attenuated calcification of cultured mouse VSMCs and aortic rings induced by calcification medium, whereas miR155 overexpression promoted VSMC calcification. Compared with wild‐type mice, miR155^−/−^ mice showed significant resistance to vitamin D3 induced vascular calcification. Protein analysis showed that miR155 deficiency alleviated the reduction of Rictor, increased phosphorylation of Akt at S473 and accelerated phosphorylation and degradation of FOXO3a in cultured VSMCs and in the aortas of vitamin D3‐treated mice. A PI3K inhibitor that suppresses Akt phosphorylation increased, whereas a pan‐caspase inhibitor that suppresses apoptosis reduced VSMC calcification; and both inhibitors diminished the protective effects of miR155 deficiency on VSMC calcification. In conclusion, miR155 deficiency attenuates vascular calcification by increasing Akt phosphorylation and FOXO3a degradation, and thus reducing VSMC apoptosis induced by calcification medium.

## 1 INTRODUCTION

Vascular calcification is a common condition encountered in advanced atherosclerotic lesions, diabetes and chronic kidney disease.^1^, ^2^, ^3^ It contributes to the mortality of these chronic diseases.^4^, ^5^ Recent advances have shown that vascular calcification is an active process rather than a degenerative, passive process at the end stage of vascular diseases. An imbalance between inducers and inhibitors has been suggested to play a critical role in the pathological process of vascular calcification.^6^ Genetic deletion of matrix Gla protein,^7^ KLOTHO^8^ or osteoprotegerin^9^ in mice elicits marked vascular calcification and osteoblastic phenotype of vascular smooth muscle cells (VSMCs), suggesting that vascular calcification is actively regulated by VSMCs that acquire osteoblast‐like properties.

MicroRNAs (miRs) are short non‐coding RNA molecules that regulate gene expression post‐transcriptionally through base pairing with mRNAs, resulting in either translational repression or mRNA degradation.^10^ They are estimated to regulate up to a third of all human genes^11^ and to play critical roles in many physiological or pathological processes, including cell differentiation, immunity development and cancer transformation. Recent studies have identified miRs as key regulators of vascular calcification by regulating the transdifferentiation of VSMCs to osteoblast‐like cells and the functional responses of other relevant cell types.^12^ miR125b,^13^ miR133,^14^ miR204^15^ and miR34a^16^ have been reported to be associated with VSMC calcification.

miR155 is encoded in the B‐cell integration cluster (*Bic*) gene^17^ and is prominently expressed in many hematopoietic cell types^18^; it has been demonstrated to be oncogenic and play a crucial role in immune response regulation.^19^, ^20^, ^21^ Previous studies from our laboratory and others showed that miR155 is also expressed in aortic tissues and plays a pro‐atherogenic role.^22^, ^23^

In the current study, we showed that miR155 is highly expressed in calcific arterial media and that miR155 deficiency significantly attenuates the calcium/phosphate overload‐induced vascular calcification in vitro, ex vivo and in vivo through activating cell survival signalling and suppressing VSMC apoptosis.

## 2 MATERIALS AND METHODS

### 2.1 Chemicals

Alizarin Red S (Cat#A5533) and cholecalciferol (Vitamin D3, Cat# C9756) were purchased from Sigma‐Aldrich. Sodium phosphate dibasic (Na_2_HPO_4_), sodium phosphate monobasic (NaH_2_PO_4_) and calcium chloride (CaCl_2_) were purchased from Fisher Scientific. 5% silver nitrate solution (Cat# 6826‐16) was purchased from RICCA Chemical Company.

### 2.2 Human vascular tissue samples

The human carotid artery samples were retrieved from patients undergoing carotid endarterectomy at Greenville Hospital in Greenville, SC, USA. The human sample collection procedure was approved by Greenville Hospital under IRB# Pro00027940. Written informed consent was universally obtained from each patient, and all study procedures conformed to the ethical standards of the Declaration of Helsinki. Calcific and nearby non‐calcific parts of arteries, or artery segments with atheroma with varied extent of calcification were snap frozen in liquid nitrogen with QIAzol™ Lysis Reagent and stored at −80°C.

### 2.3 Animals

C57BL/6J and miR155^−/−^ mice were purchased from The Jackson Laboratories (Bar Harbor, Maine) and housed in the University of South Carolina Animal Research Facility. Animal care procedures and experimental methods were in accordance with the National Institute of Health guidelines and approved by the Institutional Animal Care and Use Committee of the University of South Carolina. Mice were treated with vitamin D3 (VitD3) by intraperitoneal injection for 10 days, and then killed under anaesthesia with isoflurane inhalation for calcification analysis. miR155 transgenic (miR155tg) mice were generated in our laboratory as previously described.^24^

### 2.4 Cell isolation and culture

Mouse aortic smooth muscle cells were isolated from mouse aortas by collagenase digestion according to the published guideline^25^ and cultured in DMEM supplemented with 10% foetal bovine serum, 1% penicillin‐streptomycin, and 2 mmol/L l‐glutamine. Normal cells from passages five to ten were used for experiments. To induce calcification, cells were cultured in calcific medium containing DMEM supplemented with 5% FBS, 1% penicillin‐streptomycin, 2 mmol/L CaCl_2_ and inorganic phosphate (Pi; final concentration 2 mmol/L) for 7 days. Calcification medium was changed every other day.

### 2.5 Aortic ring culture

Mouse descending aortas were cut into 2‐3 mm rings, which were cultured in calcification medium for 7 days with medium changed every other day. At the end of the experiments, aortic rings were harvested for calcium content measurement or were embedded in OCT for frozen section preparation.

### 2.6 TUNEL assay

Analysis of apoptotic cells was performed using the terminal deoxynucleotidyl transferase‐mediated dUTP nick‐end labelling (TUNEL) staining kit following the manufacturer's instructions (Roche Diagnostics Corp., Indianapolis, IN, USA). Briefly, VSMCs grown on 8‐well chamber slides with serum‐free DMEM for 5 days or tissue sections were fixed with 4% paraformaldehyde in PBS for 20 minutes and then washed three times with PBS. After permeabilization with 0.2% Triton‐100 in PBS, the cells or tissue sections were washed three times with PBS and incubated with the TUNEL reaction mixture for 1 hour at 37°C. The nuclei were counterstained with DAPI (Roche Diagnostics Corp.). Images were taken using a Nikon DS‐Ri2 fluorescence microscope.

### 2.7 Cell proliferation and migration (wound‐healing) assay

Cells were seeded into 8‐well chamber slides at 3 × 10^4^/well and cultured in DMEM with 10% FBS for 24 hours. When the cells reached 70% confluence, the medium was replaced with serum‐free DMEM. Cells were serum‐starved for 12 hours then stimulated by PDGF‐BB (20 ng/mL; Sigma) for 24 hours. Cells were washed with PBS twice and fixed with 4% paraformaldehyde for Ki67 staining.

Cell migration was determined by wound‐healing assay. VSMCs were seeded into 6‐well plates and cultured in DMEM with 10% FBS at 37°C for 48 hours. When the cells reached confluence, the medium was replaced with serum‐free DMEM. The monolayer cells were serum‐starved for 12 hours before being wounded with a sterile cell scraper. The wounded monolayer was washed three times with standard medium to remove cell debris and cultured with serum‐free medium for 48 hours. The migration distances at 24 and 48 hours between the leading edge of the migrating cells, and the migrated cell areas were measured and analysed with Image‐Pro Plus 6.0 software (Media Cybernetics, Rockville, MD).

### 2.8 Real‐time PCR

Total RNA was extracted using QIAzol™ Lysis Reagent and RNeasy kit (Qiagen) and 1 μg of the RNA was reverse transcribed into cDNA using Bio‐Rad iScript cDNA Synthesis kit (Bio‐Rad). Gene‐specific primers were designed to flank exon junctions to ensure that all of the expected PCR products were generated from mRNA (Table S1). Real‐time PCR analysis was carried out using iQ SYBRGreen Supermix (Bio‐Rad) on a CFX96 system (Bio‐Rad). Each sample was analysed in triplicate, and target genes were normalized to the reference housekeeping gene 18s. Fold differences were then calculated for each treatment group using normalized CT values for the control. MicroRNAs were extracted using miRNeasy Mini kit (Qiagen) and were reverse transcribed by miScript‐II Reverse Transcription Kit (Qiagen). miR155 expression was detected according to the manufacturer's instructions using the miScript SYBRGreen PCR Kits (Qiagen, Valencia, CA) and miScript Primer Assays.

### 2.9 Alizarin Red S staining

The cells were washed three times with PBS and fixed by adding 1 mL of 4% formaldehyde solution for 15 minutes at room temperature and rinsed twice with ddH2O. Then, 1 mL of Alizarin Red S solution in water (40 mmol/L, pH 4.2) was added to each well, and the whole plates were kept at room temperature for 30 minutes with gentle shaking. The dye was removed, and cells were rinsed 5 times (5 minutes each time) with ddH_2_O.

To measure Alizarin Red S concentration, each well was treated with 800 μL of 10% acetic acid and incubated for 30 minutes at room temperature with shaking. Cells were scraped from the plate and transferred to a 1.5‐ml microcentrifuge tube and sealed with parafilm. After vortexing vigorously for 30 seconds, the samples were heated to 85°C for 10 minutes. Then, they were transferred on ice for 5 minutes and centrifuged at 17 000 g for 15 minutes. After centrifugation, the slurry was transferred to a new tube, and pH was adjusted to 4.1‐4.5 by adding 75 μL of 10% ammonium hydroxide. Absorbance was measured at 405 nm with a microplate reader.

### 2.10 Von Kossa staining

Cells in culture dishes or frozen sections of aorta were fixed in 10% formalin for 15 minutes at room temperature. Cells or sections were washed with distilled water three times then incubated with 5% silver nitrate for 30 minutes at room temperature. The cells or sections were exposed to ultraviolet light for 45 minutes until colour development was complete. The silver nitrate solution was removed, and the cells or sections were washed with double distilled water and photographed by microscopy (Nikon, Ds‐Ri2, Japan).

### 2.11 Calcium content quantification

Cells and tissues were decalcified with 0.6N HCl at 4°C for 24 hours. The calcium content of the HCl supernatants was determined colorimetrically using a calcium assay kit (QuantiChrom™ Calcium Assay Kit, BioAssay Systems, Hayward, WI) according to the manufacturer's recommendations. Briefly, 5 μL of the samples was transferred to a 96‐well plate. Working reagent (200 μL) was added, and absorbance was then measured at 570 nm using a microplate ELISA reader (BioTek Instruments). After decalcification, cells were washed three times with PBS and solubilized with 0.1N NaOH/0.1% SDS. The protein content was measured with a DC™ Protein Assay kit (Bio‐Rad). The calcium content of VSMCs was then normalized to the protein content, whereas that of the tissues was normalized to tissue dry weight.

For serum calcium measurement, blood samples were obtained from mice, kept on ice overnight and used for assay after centrifugation at 10 000 rpm. for 30 minutes. Serum calcium was measured using the QuantiChrom calcium assay kit (BioAssay Systems).

### 2.12 Isolation of nuclear and cytoplasm extracts

The nuclear and cytoplasm portion isolation was performed using Cell Fractionation Kit (Cell Signaling Technology, Inc). According to the instructions, VSMCs were harvested with trypsin‐EDTA and washed with cold PBS and then centrifuged at 350 *g* for 5 minutes. 500 μL Cytoplasm Isolation Buffer was added to the cell pellet to fully suspend it by vigorously vortex for 5 seconds. The suspension was kept on ice for 5 minutes and centrifuged for 5 minutes at 500 *g*. The cytoplasm portion was in the supernatant fraction, which was transferred to a pre‐chilled tube and stored on ice. The pellet fraction was resuspended in 500 μL Membrane Isolation Buffer and vortex for 15 seconds, then incubate on ice for 5 minutes and centrifuge for 5 minutes at 8000 *g*. The pellet was resuspened in 250 μL of Cytoskeleton/Nucleus Isolation Buffer (CyNIB) and sonicate for 5 seconds at 20% power 3 times, becoming the nuclear fraction.

### 2.13 Western blot analysis

Cells were lysed and protein extracted with RIPA buffer (Thermo Fisher Scientific). Then, 30 μg of total proteins was separated by 10%‐15% SDS‐polyacrylamide gel electrophoresis and transferred onto a polyvinylidene difluoride membrane (EMD Millipore). After blocking with 5% fat‐free milk solution, the protein of interest was detected by using a primary antibody and a corresponding HRP‐linked secondary antibody. The primary antibodies used were as follows: anti‐Runx2 (0.4 μg/mL), anti‐eNOS (0.5 μg/mL) from Santa Cruz Biotechnology; anti‐β‐actin (0.4 μg/mL) from Sigma‐Aldrich; anti‐osteopontin (OPN) (1 μg/mL) from Bioworld Technology; anti‐pAktS473 (1:2000), anti‐pAktT308 (1:1000), anti‐Akt (1:1000), anti‐Rictor (1:1000), anti‐RheB (1:1000), anti‐p85α (1:1000), anti‐Calponin‐1(1:1000), anti‐PARP‐1 (1:1000), anti‐Histone H3(1:2000), anti‐Bcl‐XL(1:1000), anti‐Bim(1:1000), anti‐Bax(1:1000), anti‐pS6K(1:1000), anti‐S6K(1:1000) from Cell Signaling Technology; anti‐pFOXO3a (1:2000), anti‐FOXO3a (1:1000), anti‐α‐SMA (0.5 μg/mL) from Abcam, anti‐caspase 3 (1:2000) from Cell Signaling Technology. and anti‐GAPDH (1 μg/mL) from GeneTex. The blots were developed with an enhanced chemiluminescence (ECL) reagent (Thermo Fisher Scientific) and exposed to X‐ray film to obtain optimal results in the dark room. The density of bands was quantified by Image‐Pro Plus 6.0 software.

### 2.14 Immunofluorescence

Immunofluorescence staining was performed on VSMCs plated onto chamber slides. Prior to immunostaining, cells were washed twice with PBS, fixed with 4%paraformaldehyde for 15 minutes and permeabilized for 10 minutes using 0.05% Triton^®^ 100. VSMCs were incubated for 1 hours at room temperature with 5% BSA to block nonspecific binding. Cells were then incubated overnight with rabbit anti‐ki67 and mouse anti‐SMA antibodies and for 1 hours with the appropriate secondary antibody conjugated to Alexa‐488 or Alexa‐594 (Invitrogen). DAPI was used to counterstain the nuclei. Samples were covered with DABCO mounting media (Sigma‐Aldrich), overlaid with coverslips and examined under a Nikon DS‐Ri2 fluorescence microscope. The percentage of ki67 and SMA double positive cells were normalized to all SMA‐positive cells using Image‐Pro Plus 6.0 software.

### 2.15 Induction of vascular calcification in mice

WT or miR155^−/−^ C57BL/6J mice were used for vascular calcification induction by administration of Vitamin D3 (VitD3). As our mice could not survive more than 3 days using the previously described VitD3 solution,^26^ we modified the VitD3 solution with a different solubilization protocol. VitD3 was first dissolved in absolute ethanol (5 mg per 40 μL) and then diluted to a concentration of 3.75 mg/mL with highly refined olive oil (Sigma‐Aldrich). Stock solutions of VitD3 were prepared fresh for each 3‐day injection cycle and then placed in foil wrapped containers and stored at 4°C. The mice were intraperitoneally injected with VitD3 (500 000 IU/kg/d in 100 μL) or vehicle (100 μL olive oil per day) for 10 days. On the 14th day after injection, blood and aorta tissues were harvested for analysis.

### 2.16 siRNA transfection

To knock down Akt or caspase 3 (Cas3) in VSMCs using siRNAs, mouse VSMCs were transfected in 24‐well plates with control siRNA (siCon, Invitrogen, Cat #12935‐300), Akt1 siRNA (siAkt, Invitrogen, MSS289605), or Caspase 3 siRNA (siCas3, Invitrogen, MSS202635) using Lipofectamine™ RNAiMAX following the manufacturer's instructions. Two days after the transfection, some wells of cells were collected to determine the knockdown efficiency, the other wells of cells were treated with CM for 7 days to induce calcification.

### 2.17 Statistical analysis

All data were expressed as the mean ± standard error of the mean (SEM). Statistical analyses were performed using GraphPad Prism 6.0 (GraphPad Software). Normal probability plot or Kolmogorov‐Smirnov test was performed for both groups of data to ensure the validity of normality assumption. Statistical differences were determined by a Student's *t* test for comparisons between two groups. A one‐way analysis of variance (ANOVA) followed by Bonferroni's multiple comparison tests was used for comparison among multiple groups. Correlations were assessed by Pearson correlation analysis given the data fulfilling Gaussian distribution. *P* values smaller than .05 were considered to indicate statistical significance.

## 3 RESULTS

### 3.1 miR155 is highly expressed in human calcified atherosclerosis plaques

**Figure 1:**
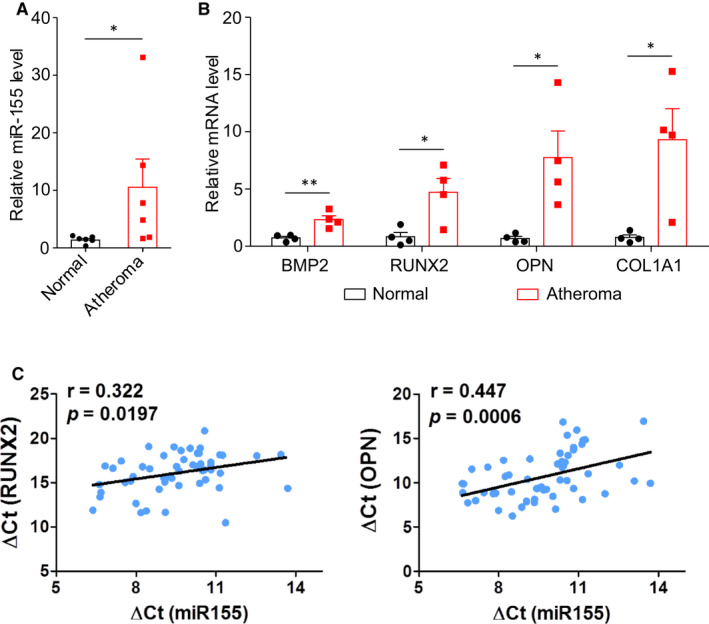
Expression levels of miR155 and osteochondrogenic genes in human carotid artery tissue. The expression levels of miR155 (A) and osteochondrogenic genes including Bone morphogenetic protein 2 (BMP2), Runt‐related transcription factor 2 (RUNX2), Osteopontin (OPN) and alpha‐1 type I collagen (COL1A1) (B) in human carotid arterial wall tissues were assessed by qPCR (N = 6 for each group). Two‐tailed Student's *t* test; *P < .05. C. The correlation between the expression levels of miR155 and those of RUNX2 and OPN in human carotid artery segments with atheroma (Pearson correlation analysis)

We first analysed the expression levels of miR155 in human calcific atheromas from patients receiving carotid endarterectomy. We found miR155 is highly expressed in calcific carotid atheromas compared with adjacent non‐calcific media (Figure 1A). Moreover, the osteogenic markers, including BMP2, RUNX2, OPN and COL1A1, were increased in calcific atheromas (Figure 1B). To confirm the relationship between miR155 and osteogenic markers, we collected 56 additional human carotid samples and measured the expression of miR155 and osteogenic genes in the carotid artery segments with calcified atheroma using qPCR. We found that miR155 expression is positively correlated with the expression levels of RUNX2 and OPN (Figure 1C). These data suggest a role of miR155 in arterial calcification, indicating that miR155 may modulate the biology of vascular smooth muscle cells (VSMCs), which are a major component in human atherosclerotic lesions.

### 3.2 miR155 deficiency affects survival and migration ability of VSMCs

**Figure 2:**
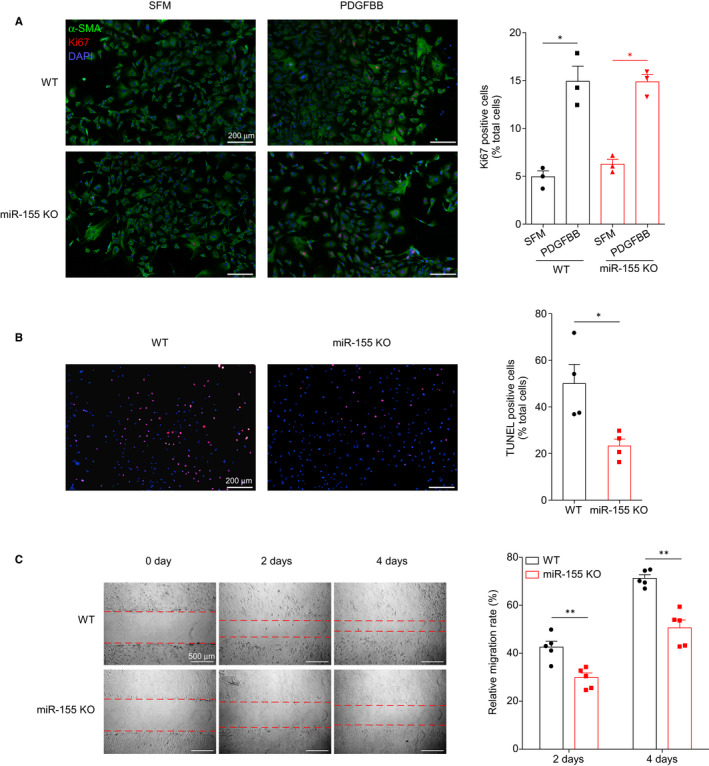
miR155 deficiency promotes VSMC survival and inhibits VSMC migration. A, Proliferation rates of wild‐type (WT) and miR155^−/−^ VSMCs with or without PDGFBB treatment were assessed by Ki67 immunostaining. Relative proliferation rate is normalized to total cell numbers. Scale bar, 200 μm. B, Serum starvation induced apoptosis of WT and miR155^−/−^ VSMCs was assessed by TUNEL staining. Relative apoptotic rate is normalized to total cell numbers. Scale bar, 200 μm. C, Migration ability of WT and miR155^−/−^ VSMCs was assessed by wound‐healing test. Scale bar, 500 μm. Data are shown as mean ± SEM of triplicates and are representative of three independent experiments. N = 6; *P < .05, two‐tailed Student's *t* test

The effects of miR155 on VSMC function have not been examined. First, we found VSMCs express miR155 at 40% levels of peritoneal macrophages in mice (Figure S1). We then investigated how miR155 deficiency affects VSMC functions. The proliferation, survival and migration ability of miR155 deficient VSMCs were examined. We found that miR155 deficiency did not affect the baseline or PDGF‐BB induced proliferation of VSMCs (Figure 2A). However, miR155 deficiency improved cell survival upon serum starvation (Figure 2B) and inhibited VSMC migration (Figure 2C) in vitro.

### 3.3 miR155 expression modulates VSMC and aortic calcification

**Figure 3:**
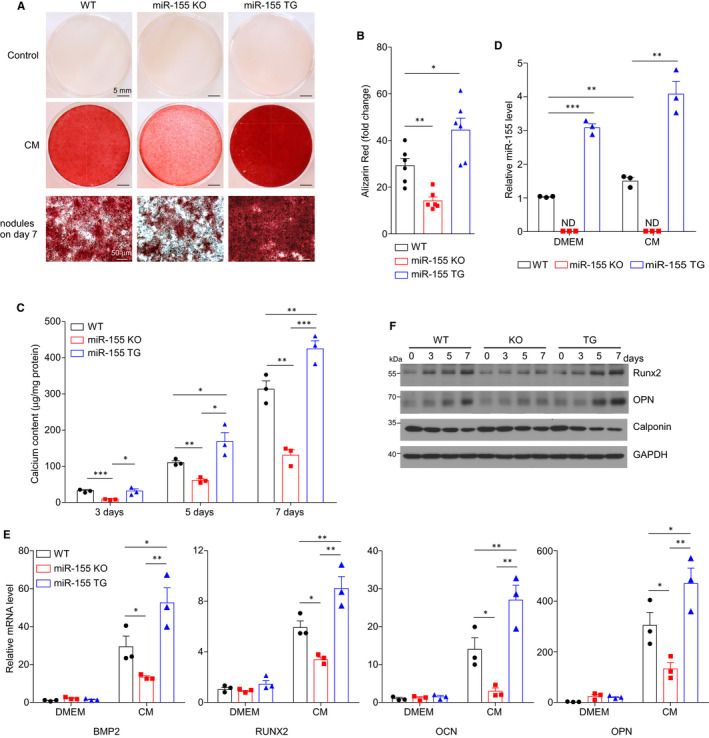
miR155 modulates VSMC calcification in vitro. A, Calcium deposition of VSMCs was visualized by Alizarin Red S (pH 4.2) staining. Representative microscopy images of Alizarin Red S staining of VSMCs isolated from WT, miR155^−/−^ (KO) or miR155tg (TG) mice. B, Quantification of Alizarin Red staining measured with a microplate reader and normalized to total protein content. Fold changes compared with corresponding control are calculated. C, Calcium content of VSMCs was assessed calorimetrically using a calcium assay kit. D, The miR155 levels in VSMCs isolated from WT, miR155^−/−^ and miR155tg mice were determined by qPCR. E, The mRNA levels of osteochondrogenic genes including BMP2, RUNX2, OPN and Osteocalcin (OCN) in VSMCs were assessed by qPCR. F, Protein levels of RUNX2, OPN and Calponin were assessed by Western blot; the quantification is shown in Figure S2. CM: calcific medium containing 2 mmol/L Ca^2+^ &amp;Pi^+^. Data are shown as mean ± SEM of triplicates and are representative of three independent experiments. N = 3‐5; *P < .05, **P < .01, ***P < .001; two‐tailed Student's *t* test

To induce VSMC calcification, mouse aortic smooth muscle cells from WT, miR155^−/−^ or miR155tg mice were cultured with calcification medium containing 2 mmol/L inorganic phosphate and 2 mmol/L CaCl_2_. Alizarin Red S staining showed that miR155 deficient VSMCs have less, whereas VSMCs from miR155tg mice have more calcium deposition than WT VSMCs (Figure 3A‐C). Compared with WT VSMCs, VSMCs from miR155tg mice had a threefold increase in miR155 expression, and miR155 expression was significantly increased (~1.5 fold) upon incubation with calcification medium in WT VSMCs; whereas miR155 expression is not detectable in miR155^−/−^ VSMCs (Figure 3D). The mRNA levels of osteogenic markers BMP2, RUNX2, OPN and OCN were lower in miR155 deficient VSMCs and higher in miR155tg VSMCs than in WT VSMCs after the cells were treated for 7 days (Figure 3E). We also analysed the protein levels of RUNX2 and OPN in VSMCs, and found that miR155 deficiency decreased, whereas miR155 overexpression increased the levels of both proteins in VSMCs treated with calcification medium (Figure 3F and Figure S2).

**Figure 4:**
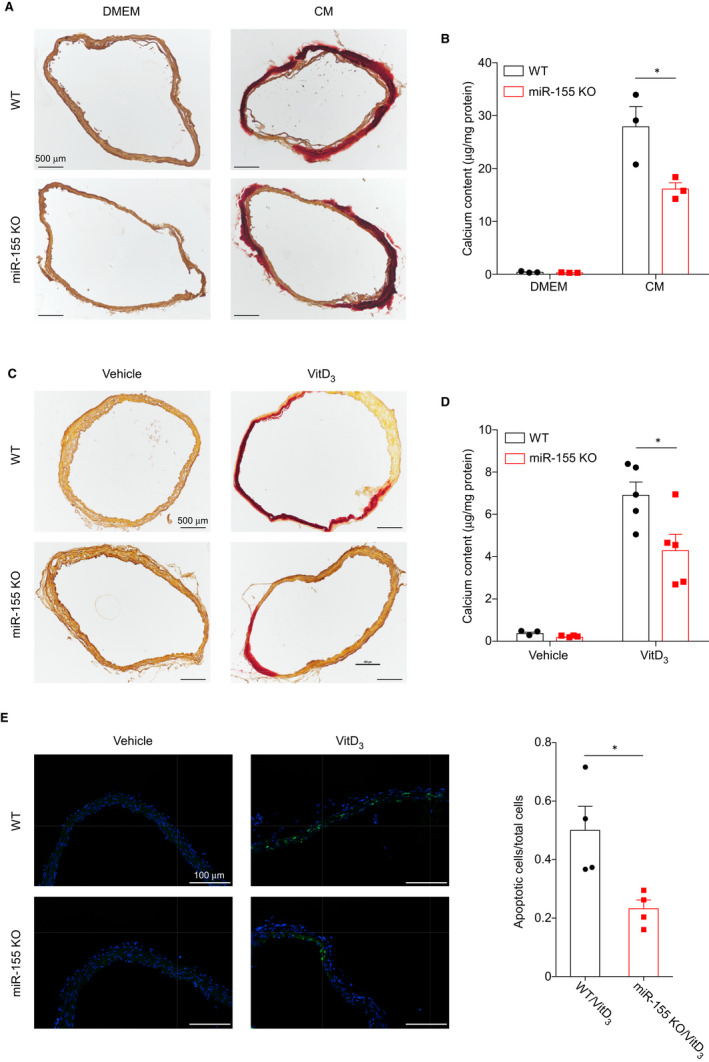
miR155 deficiency attenuates aortic calcification ex vivo and in vivo. A, Calcium deposition in cultured aortic rings from WT or miR155^−/−^ mice was visualized by Alizarin Red S staining. B, Quantification of calcium deposition was performed calorimetrically using a calcium assay kit. C, Calcium deposition in aortic arch from WT or miR155^−/−^ mice receiving vitD3 injection was visualized by Alizarin Red S staining. (N = 5 for each group). D, Quantification of calcium deposition in aortic arches. Scale bar, 100 μm. Data are shown as mean ± SEM of triplicates and are representative of three independent experiments. *P < .05. E, Representative images (Left) and quantification (Right) of TUNEL staining of aortic arches from WT or miR155^−/−^ mice treated with vehicle or vitD3. (N = 5 for each group). *P < .05; two‐tailed Student's *t* test

To further investigate the effects of miR155 on vascular calcification, we measured the degree of calcification in aortic rings cultured with calcification medium, as well as in vivo aortic calcification induced by VitD3 in male mice. We only used male mice because literatures have shown sex difference in vascular calcification and suggested that oestrogen protects females from vascular calcification.^27^, ^28^, ^29^ Our data showed miR155 deficiency attenuated calcium deposition both in cultured aortic ring (Figure 4A‐B and Figure S3A) and in aortas of 20‐week‐old male mice (Figure 4C‐D and Figure S3B). In agreement with the in vitro data, Rux2 protein levels in the aortas of miR155^−/−^ mice are significantly lower than those in the aortas of WT mice (Figure S3C). It is noteworthy that the serum calcium levels in VitD3 treated miR155 deficient mice were slightly but significantly higher than those in WT mice treated with VitD3 (Figure S3D), suggesting that the reduced aortic calcification was not because of reduced serum calcium levels. TUNEL staining showed the apoptotic cell number was lower in the aortas of miR155^−/−^ mice than in those of WT mice (Figure 4E).

### 3.4 miR155 affects Akt activation during VSMC calcification

**Figure 5:**
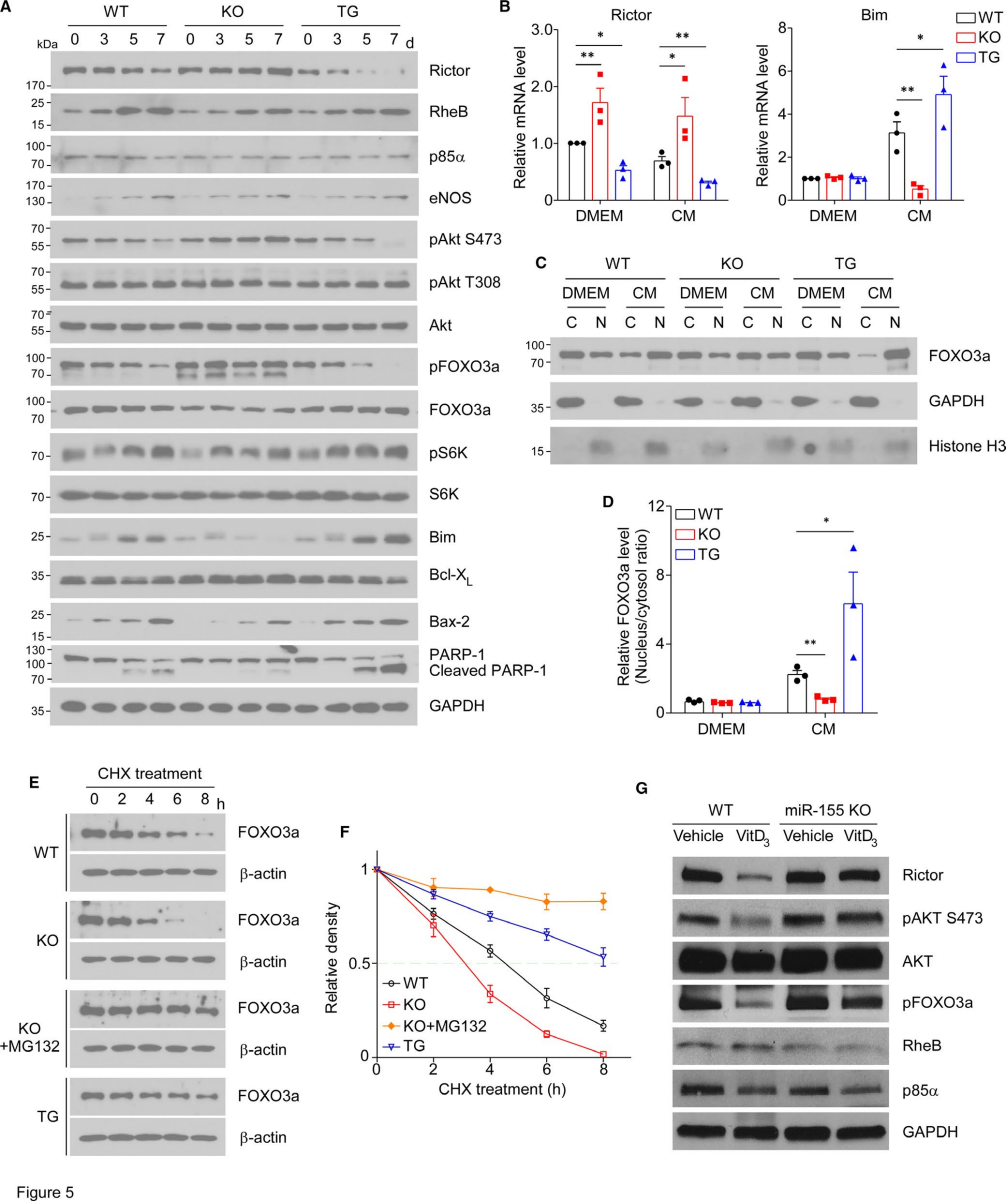
miR155 altered Akt‐FOXO3a signalling in vitro and in vivo. A, Confluent aortic VSMCs isolated from WT, miR155^−/−^ or miR155tg mice were cultured in calcification medium (CM, 2 mmol/L phosphate/2 mmol/L calcium) for 0 (control), 3, 5 or 7 d, and total cellular lysates were subjected to Western blot analysis. Quantification is shown in Figure S4A. B, The mRNA levels of Rictor and Bim in cells treated with DMEM or CM for 7 d were quantified by qPCR (normalized to GAPDH). Data are presented as mean ± SD (N = 3); one‐way ANOVA followed by Bonferroni's multiple comparison tests; *P < .05, **P < .01. C, D, VSMCs isolated from WT, miR155^−/−^ or miR155tg mice were treated with DMEM or CM for 7 d, then cell lysates were harvested and used to extract proteins from cytoplasm and nucleus. Western blot bands were quantified using Gel‐Pro Analyzer software and presented as fold changes of FOXO3a level in nucleus compared with cytosol. GAPDH and Histone H3 were blotted to demonstrate the purity of each fraction. Data are presented as mean ± SD (N = 3); one‐way ANOVA followed by Bonferroni's multiple comparison tests; *P < .05, **P < .01. E, VSMCs were treated by calcification medium for 7 d and then incubated with 50 µg/mL cycloheximide (CHX, a protein synthesis inhibitor) for indicated time. Some miR155^−/−^ VSMCs were pretreated with 50 µmol/L MG132 (a proteasome inhibitor that inhibits protein degradation). FOXO3a was detected by Western blot. F, Quantitative analysis of FOXO3a protein levels was shown. G, Protein levels of pAkt, pFOXO3a, Rictor and RheB in aortic tissues of mice treated with VitD3 (in Figure 4C) were assessed by Western blot. Quantification of their relative protein levels normalized to total Akt or GAPDH is shown in Figure S4B

Rapamycin‐insensitive companion of mTOR (Rictor) and Ras homolog enriched in brain (Rheb) have been recently reported as the direct targets of miR155 in cancer cells,^30^, ^31^ and Rictor has been shown to play a critical role in mTORC2 induced Akt phosphorylation. To examine the potential mechanisms by which miR155 modulates VSMC apoptosis and vascular calcification, we analysed Rictor‐Akt signalling and found that miR155 deficiency significantly increased whereas miR155 overexpression significantly decreased the Rictor protein levels and phosphorylation of Akt at S473 in VSMCs treated with calcification medium, without an effect on RheB (Figure 5A and Figure S4A). We also detected the levels of apoptosis‐related proteins, including Bim, Bcl‐X_L_, Bax‐2 and PARP‐1. It is shown that miR155 deficiency reduced the pro‐apoptotic protein Bim, Bax2 and cleaved PARP‐1 whereas increased the anti‐apoptotic protein Bcl‐X_L_; conversely, miR155 overexpression increased Bim and cleaved PARP‐1 and decreased Bcl‐X_L_ (Figure 5A and Figure S4A). qPCR analysis confirmed that Rictor and Bim were regulated by miR155 expression at mRNA levels in opposite directions, especially in the presence of calcification medium (Figure 5B). The phosphorylated FOXO3a, a downstream target of Akt, was dramatically suppressed by calcification medium treatment. This suppressive effect was significantly diminished in miR155 deficient VSMCs whereas enhanced in miR155‐overexpressing VSMCs (Figure 5A and Figure S4A). As phosphorylation of FOXO3a leads to its translocation from nucleus to cytoplasm and degradation, we measured the ratio of nuclear and cytoplasmic FOXO3a protein in VSMCs and found that, indeed, miR155 deficiency reduced whereas miR155 overexpression increased the nucleus/cytosol ratio of FOXO3a protein (Figure 5C‐D). A time course experiment further confirmed that miR155 deficiency enhanced whereas miR155 overexpression attenuated FOXO3a degradation in VSMCs (Figure 5E‐F). Because FOXO3a directly controls the transcription of Bim, a pro‐apoptotic protein, this agrees with that data that both mRNA and protein levels of Bim were positively correlated with nuclear and total FOXO3a levels. We further examined the levels of these proteins in aortic tissues from the mice treated by VitD3 to induce aortic calcification; we found significant increases of Rictor, pAKT S473 and pFOXO3a in the aortas of VitD3‐treated miR155^−/−^ mice than those in WT mice, whereas the levels of RheB and PI3K subunit P85α, a reported target protein of miR155 in B‐lymphocytes, showed no difference between WT and miR155^−/−^ aortas (Figure 5G and Figure S4B).

### 3.5 miR155 modulates VSMC calcification by promoting apoptosis

**Figure 6:**
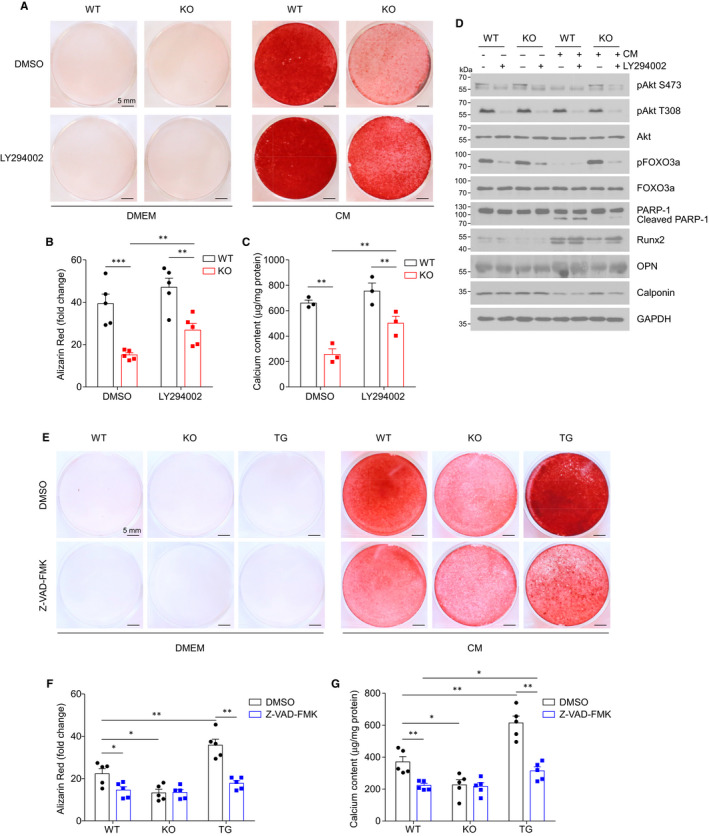
miR155 modulates VSMC calcification by enhancing apoptosis. A, VSMCs isolated from WT or miR155^−/−^ mice were treated with LY294002 (50 μmol/L) for 1 h and then cultured in CM. Treatment was repeated (LY294002 treatment for 1 h followed by CM culture) every 2 d, and cells were stained after 7 d with Alizarin Red S (representative images are shown). B, Quantified Alizarin Red S as extracted from each culture well treated with CM and read on a microplate reader. Data shows mean ± SEM, N = 6 each group; *P < .05, **P < .01, ***P < .001, two‐tailed Student's *t* test. C, Calcium contents of VSMCs treated with CM and with or without LY294002 were assessed calorimetrically using a calcium assay kit. Data are shown as mean ± SEM; N = 6; *P < .05, **P < .01, ***P < .001, two‐tailed Student's *t* test. D, The effect of LY294002 on phosphorylation of Akt and FOXO3a as well as protein levels of Runx2, OPN, Calponin and cleaved PARP‐1 were determined by Western blot analysis. The quantification is shown in Figure S5. E, VSMCs isolated from WT, miR155^−/−^ or miR155tg mice were cultured with CM with or without Z‐VAD‐FMK (50 μmol/L) for 7 d and stained with Alizarin Red S for calcium deposition (representative images are shown). F, Quantified Alizarin Red S as extracted from each culture well treated with CM and read on a microplate reader. Data shows mean ± SEM, N = 6; *P < .05, **P < .01, two‐tailed Student's *t* test. G, Calcium contents of VSMCs treated with CM and with or without Z‐VAD‐FMK were assessed calorimetrically using a calcium assay kit. Data are shown as mean ± SEM; N = 6; *P < .05, **P < .01, two‐tailed Student's *t* test

The above results suggest that miR155 may modulate VSMC calcification by regulating Akt signalling and apoptosis. To further confirm the role of Akt signalling in mediating miR155’s effects on VSMC calcification, we used a PI3K inhibitor, LY294002, which suppresses Akt phosphorylation, to treat VSMCs that were subjected to calcification induction. As shown in Figure 6A‐C, LY294002 significantly increased calcification in miR155^−/−^ VSMCs (77.4% increase in Alizarin staining and 95.9% in calcium content), but only slightly and not significantly increased calcification in WT cells (only 19.6% in Alizarin staining and 14.3% in calcium content). Western blot analysis showed that whereas LY294002 did not significantly alter the protein levels or phosphorylation in WT VSMCs, it significantly reduced pAkt S473, pFoxO3 and Calponin and increased RunX2, Opn and cleaved PARP‐1 in miR155^−/−^ VSMCs (Figure 6D and Figure S5). To further confirm the role of Akt in miR155‐enhanced VSMC calcification, we used siRNA to knockdown Akt1 in WT or miR155^−/−^ VSMCs and examined the impact on calcification. Akt1 protein levels were reduced substantially by siRNA (Figure S6A); both WT and miR155^−/−^ VSMCs displayed significantly increased CM‐induced calcification in the cells with Akt1 knockdown, and the increase was more robust in miR155^−/−^ cells (Figure S6B).

Lastly, we used a pan‐caspase inhibitor, Z‐VAD‐FMK to treat VSMCs upon calcification induction by calcification medium. The results showed that apoptosis inhibition significantly reduced CM‐induced calcification of both WT and miR155‐overexpression VSMCs and diminished the difference of calcification between WT, miR155^−/−^ and miR155‐overexpressing VSMCs (Figure 6E‐G). In addition, when Caspase 3 was knocked down by siRNA (Figure S6C), CM‐induced calcification was significantly decreased in WT VSMCs, and only slightly reduced in miR155^−/−^ cells (Figure S6D).

## 4 DISCUSSION

In this study, we demonstrated that miR155 deficiency attenuates whereas miR155 overexpression enhances vascular calcification induced by high levels of calcium and phosphate. We provided evidence that miR155 modulates vascular calcification via regulating Akt activation, FOXO3a degradation and therefore VSMC apoptosis in high calcium and high phosphate conditions. Our study sheds new insights into the role of miR155 in vascular biology.

Our data from human tissues showed that miR155 was increased in calcified carotid atheroma and that miR155 expression levels were positively correlated with the expression levels of RUNX2 and OPN, two osteogenic markers, in human carotid atheroma. These data suggest that miR155 might be involved in arterial calcification, because these osteogenic genes have been shown to associate with osteochondrogenic transdifferentiation of VSMCs.^32^ Consistently, in the calcification medium‐induced VSMC calcification cell culture model and the VitD3 induced aortic calcification mouse model, miR155 deficiency attenuated VSMC or aortic calcification, respectively; and miR155 overexpression increased calcification medium‐induced VSMC calcification. These data suggest that inhibition of miR155 may be a novel therapeutic approach for vascular calcification.

miR155 regulates various functions of cells. Sustained expression of miR155 leads to a proliferative disorder in hematopoietic stem cells associated with acute myeloid leukaemia and protects B cells from apoptosis.^33^, ^34^ The opposite pro‐apoptotic and pro‐autophagy effects of miR155 were reported in hepatocytes, vascular endothelial cells and non‐hematopoietic cancer cells.^31^, ^35^, ^36^ These data suggest that the effects of miR155 are cell type‐ and context‐dependent. Similar to those reports from non‐hematopoietic cell studies, our data demonstrated that miR155 deficiency protects VSMCs from apoptosis but inhibits VSMC migration. Previous in vitro and in vivo studies have shown that mineral imbalance including increased calcium and phosphate, induces VSMC apoptosis and vesicle release, resulting in mineral nucleation preceding the development of vascular calcification.^37^, ^38^, ^39^ Studies have shown that VSMCs release calcium in vesicle form in response to calcium overload to prevent apoptosis and that these vesicles are loaded with calcification inhibitors, including fetuin‐A and MGP, that act to limit their calcification potential.^40^, ^41^ Our data suggest that miR155 deficiency may enhance the VSMC ability to release calcium in response to VitD3 induced calcium overload and promote their survival. Apoptosis has been shown to increase the local concentrations of calcium, and with local elevation in calcium levels induces further VSMC death, and calcification.^42^ In our vascular calcification mouse model, we found that miR155 deficiency reduced the arterial cell apoptosis which is consistent with the in vitro anti‐apoptosis effect of miR155 deficiency. Interestingly, a previous study showed that miR155 suppresses autophagy in chondrocytes and contributes to the autophagy defects in osteoarthritis.^43^ As autophagy usually serves as a survival mechanism against apoptosis, it is worth further investigating if miR155 also acts as an autophagy suppressor in VSMCs and thus promotes their apoptosis under calcification‐inducing conditions.

Many signalling pathways including BMP signalling and Wnt signalling are involved in the pathogenesis of vascular calcification.^4^ Regulation of vascular calcification by Akt signalling has been recently examined. Heath et al revealed that loss of PTEN, a negative regulator of Akt, induced Akt activation and inhibited FOXO1/3 in VSMCs and promoted diabetic and atherosclerotic vascular calcification.^44^, ^45^ Interestingly, our data showed an opposite role of Akt activation and FOXO3a degradation in vascular calcification; miR155 deficiency increased Akt activation and FOXO3a phosphorylation/degradation and thus inhibited vascular calcification in our in vitro high calcium/phosphate load‐induced and in vivo Vit‐D3‐induced calcification models. We believe the different models may have contributed to the opposite outcome of Akt activation and FOXO3a degradation in vascular calcification. In our current study, we used a high inorganic calcium/phosphate induced calcification model instead of a mild osteogenic medium‐induced model in vitro, and a short‐term in vivo intensive vascular calcification model instead of a moderate long‐term diabetic or atherosclerotic vascular calcification model. Our in vivo study showed a decreased Akt activation in calcific aorta tissue, whereas in previous reports Akt activation was increased in calcific aorta tissue in a different mouse model.^44^, ^45^ Our model is similar to clinical vascular calcification occurring in chronic renal disease characterized by a high calcium/phosphate load and VSMC apoptosis in the arterial wall. It is noteworthy that in Heng and colleagues’ model, Akt activation did not affect VSMC survival.^44^ Our data suggest that Akt activation might play a different role in vascular calcification caused by different pathological insults. Runx2 is a key transcriptional regulator required for vascular calcification.^46^, ^47^, ^48^, ^49^ In atherosclerotic vascular calcification, Deng et al showed that Akt activation and the resulting FOXO3a phosphorylation increase RUNX2 expression.^45^ On the contrary, our data showed that high calcium/phosphate load‐induced expression of RUNX2 was accompanied by reduced Akt activation and FOXO3a phosphorylation/degradation. As the main effect of Akt‐mediated FOXO3a phosphorylation is preventing cell apoptosis, and Akt does not directly induce RUNX2 expression,^50^ we speculate that in our VSMC/vascular calcification models, miR155 deficiency mediated Akt activation mainly acts on cell survival. Indeed, in our cell culture model, when a PI3K inhibitor was used to suppress Akt activation, both apoptosis and calcification of VSMCs increased; and knockdown of Akt1 by siRNA significantly enhanced CM‐induced calcification of VSMCs. And when apoptosis was inhibited by a pan‐caspase inhibitor or siRNA, VSMC calcification was significantly reduced and the differences of calcification between WT, miR155^−/−^ and miR155‐overexpressing cells diminished. Nevertheless, it is intriguing to further elucidate the model‐dependent role of Akt/FOXO3a in vascular calcification.

Although miR155 is expressed in other cell types such as endothelial cell and macrophages in the vasculature, this study is focused on the role of miR155 in VSMC calcification using in vitro cultures of VSMCs and aortic rings in addition to vitD3‐induced calcification mouse model, because VSMCs are the major cell type contributing to arterial calcification. It would be interestingly in future to examine the cell type‐specific role of miR155 in vascular calcification using cell type‐specific miR155 knockout or overexpression mouse models.

In summary, on the basis of our data, we propose a molecular mechanism by which that miR155 regulates high calcium/phosphate load‐induced vascular calcification (Figure S7). In high calcium/high phosphate condition, miR155 expression is increased in VSMCs. miR155 suppresses the activation of Akt through depressing Rictor/mTORC2, leading to reduced FOXO3a phosphorylation and degradation. Accumulation of FOXO3a in nucleus activates the transcription of pro‐apoptotic proteins such as Bim and thus enhances VSMC apoptosis. Apoptotic VSMCs subsequently facilitates vascular calcification. Inhibition of VSMC apoptosis through targeting miR155 expression or function in VSMCs may be developed as an approach to halt vascular calcification in the conditions with increased serum calcium/phosphate load such as chronic kidney diseases.

## CONFLICT OF INTEREST

None declared.

## AUTHOR CONTRIBUTIONS

**Yong Li:** Data curation (equal); formal analysis (equal); investigation (equal); methodology (equal); writing‐review and editing (supporting). **Wei Sun:** Conceptualization (equal); data curation (equal); formal analysis (equal); investigation (equal); writing‐original draft (equal). **Fatma Saaoud:** Data curation (supporting); methodology (supporting). **Yuzhen Wang:** Data curation (supporting); investigation (supporting); methodology (supporting). **Quanyi Wang:** Investigation (supporting); methodology (supporting). **Johnie Hodge:** Investigation (supporting); methodology (supporting); writing‐review and editing (supporting). **Yvonne Hui:** Investigation (supporting); methodology (supporting). **Sophia Yin:** Investigation (supporting); methodology (supporting). **Susan M. Lessner:** Resources (supporting); writing‐review & editing (supporting). **Xiangqing Kong:** Methodology (supporting); Writing‐review and editing (supporting). **Daping Fan:** Conceptualization (equal); funding acquisition (lead); investigation (equal); project administration (lead); resources (lead); writing‐original draft (equal); writing‐review and editing (lead).

## Supporting information

Supplementary Material
