# Supplementary material for: MiR155 modulates vascular calcification by regulating Akt‐FOXO3a signalling and apoptosis in vascular smooth muscle cells
Source: J Cell Mol Med. 2020 Nov 18;25(1):535–48. doi: 10.1111/jcmm.16107 (PMC7810936; doi:10.1111/jcmm.16107)
Supplement: Supplementary file 1 — Supplementary Material [file JCMM-25-535-s001.docx]

**Supplementary Information**

**Supplementary Table S1: Primers used for the quantitative PCR**

| Primers |  | Sequence 5’- 3’ |
| --- | --- | --- |
| *mBMP2* | Forward | GGGACCCGCTGTCTTCTAGT |
|  | Reverse | TCAACTCAAATTCGCTGAGGAC |
| *mRUNX2* | Forward | GACTGTGGTTACCGTCATGGC |
|  | Reverse | ACTTGGTTTTTCATAACAGCGGA |
| *mOCN* | Forward | CTGACCTCACAGATCCCAAGC |
|  | Reverse | TGGTCTGATAGCTCGTCACAAG |
| *mCOL1A1* | Forward | GCTCCTCTTAGGGGCCACT |
|  | Reverse | CCACGTCTCACCATTGGGG |
| *mOPN* | Forward | AGCAAGAAACTCTTCCAAGCAA |
|  | Reverse | GTGAGATTCGTCAGATTCATCCG |
| *mRICTOR* | Forward | ACTGACGCCAAGCAGGTTTA |
|  | Reverse | AGCGCTGGAGGGTATTGTGA |
| *mBIM* | Forward | GTCGTTCGATCGGCGCAA |
|  | Reverse | GGCTCCTGTCTTGCGGTT |
| *m18S* | Forward | CGCGGTTCTATTTTGTTGGT |
|  | Reverse | AGTCGGCATCGTTTATGGTC |
| *hBMP2* | Forward | ACCCGCTGTCTTCTAGCGT |
|  | Reverse | TTTCAGGCCGAACATGCTGAG |
| *hRUNX2* | Forward | TGGTTACTGTCATGGCGGGTA |
|  | Reverse | TCTCAGATCGTTGAACCTTGCTA |
| *hOPN* | Forward | CTCCATTGACTCGAACGACTC |
|  | Reverse | CAGGTCTGCGAAACTTCTTAGAT |
| *hCOL1A1* | Forward | GAGGGCCAAGACGAAGACATC |
|  | Reverse | CAGATCACGTCATCGCACAAC |

Notes: *m*, mouse; *h*, human. *BMP2*, bone morphogenetic protein 2; *RUNX2*, runt-related transcription factor 2; *COL1A1*, Collagen Type I Alpha 1; *OCN*, osteocalcin; *OPN*, osteopontin; *ON*, secreted acidic cysteine rich glycoprotein.

**Supplementary Figures**

**Supplementary Figure S1.** qPCR measurement of miR155 expression in peritoneal macrophages and vascular smooth muscle cells from same WT C57Bl/6J mice (n=3).


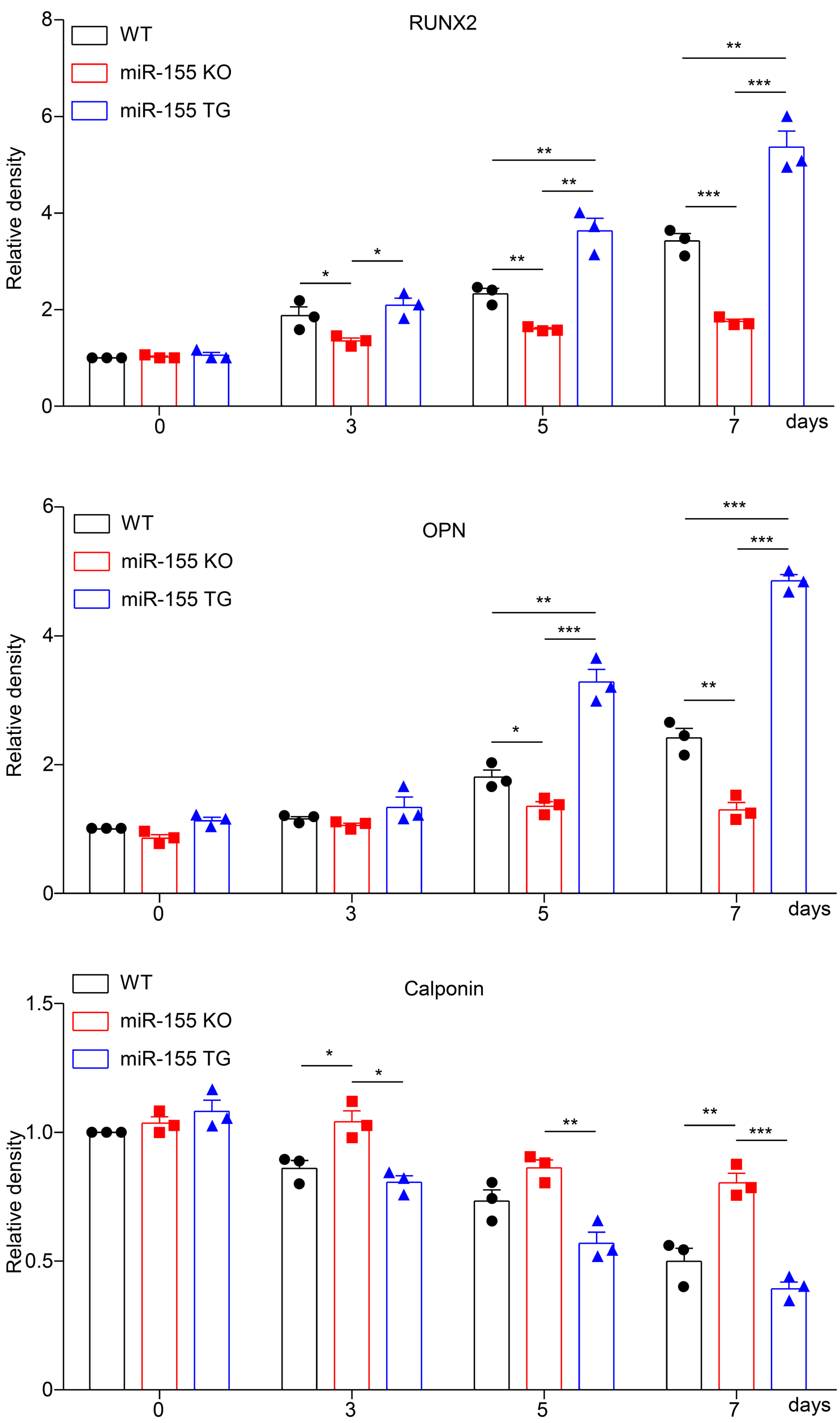


**Supplementary Figure S2.** Quantification of the relative protein levels of RUNX2, OPN and Calponin in Fig. 3F. The intensities of the indicated protein bands were normalized to GAPDH bands. Data are shown as mean±SEM of triplicates and are representative of three independent experiments. N=3; two-tailed Student *t*-test; *p<0.05, **p<0.01, ***p<0.001.





**Supplementary Figure S3.** **miR155 deficiency attenuates aortic calcification *ex vivo* and *in vivo*.** A. Calcium deposition in cultured aortic rings from WT or miR155^-/-^ mice treated with DMEM or calcification medium was visualized by Von Kossa staining. B. Calcium deposition in aortas of WT or miR155^-/-^ mice treated with vehicle or VitD3 was visualized by Von Kossa staining. C. Protein levels of Runx2 in aorta tissues of mice with or without VitD3 treatment were assessed by western blotting. The relative protein levels are normalized to GAPDH. Data are shown as mean±SEM. D. Serum calcium concentrations were measured in WT or miR155^-/-^ mice treated with vehicle or vitD3. N=5; two-tailed Student *t*-test; *P<0.05, **P<0.01.


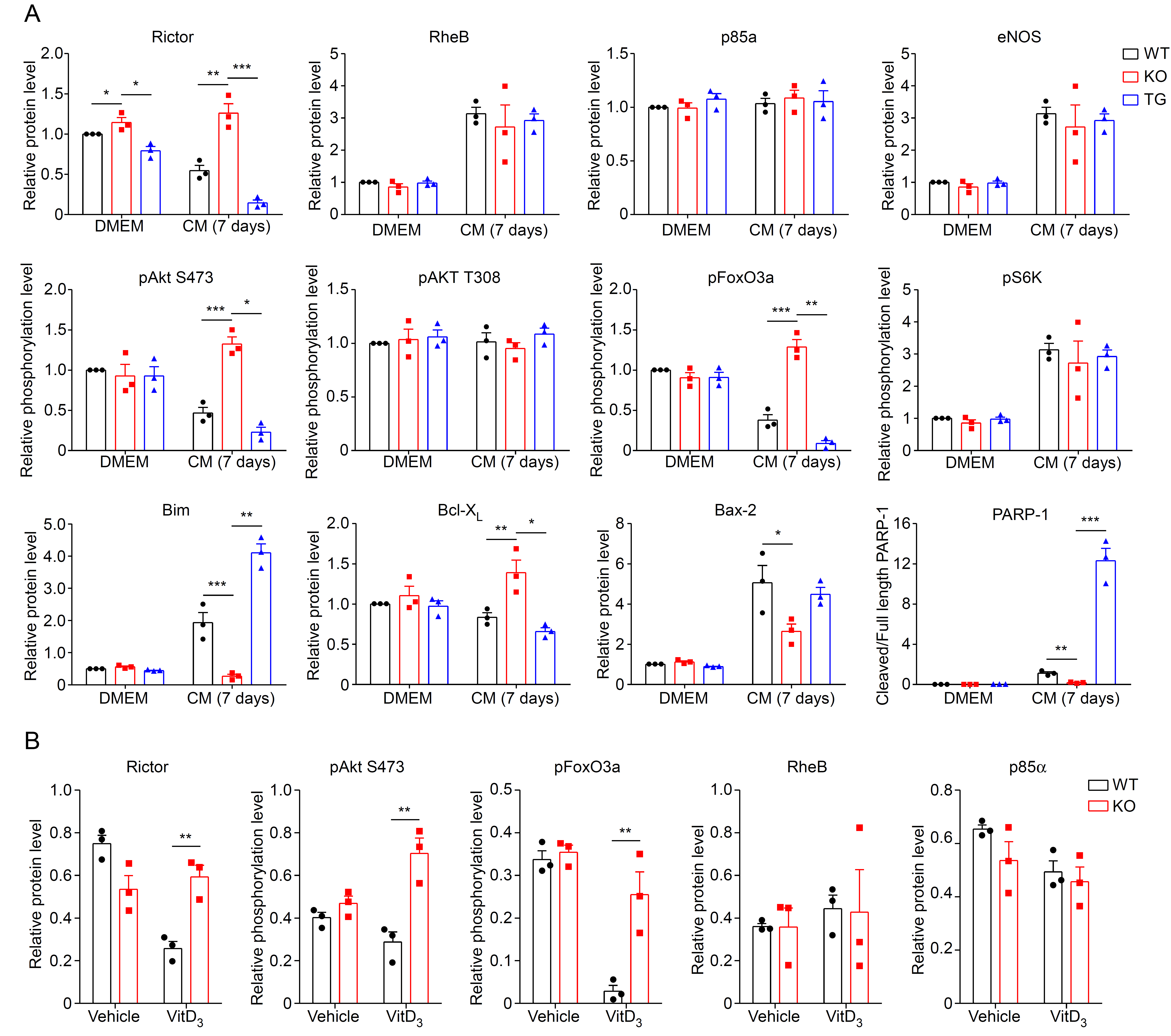


**Supplementary Figure S4.** A. Quantification of the data shown in Fig 5A. Relative fold changes of protein levels or phosphorylation levels were determined by densitometry and normalized to GAPDH or its total protein level respectively, Data are presented as mean±SEM (n=3); one-way ANOVA followed by by Bonferroni’s multiple comparison tests; ^#^p<0.05, vs WT (DMEM); *p<0.05, **p<0.01, ***p<0.001 vs WT (CM, 7 days); B. Quantification of the data shown in Fig. 5G. The relative protein levels are normalized to total Akt or GAPDH. Data are shown as mean±SEM. N=5; two-tailed Student’s *t*-test; **p<0.01 vs WT (VitD3).


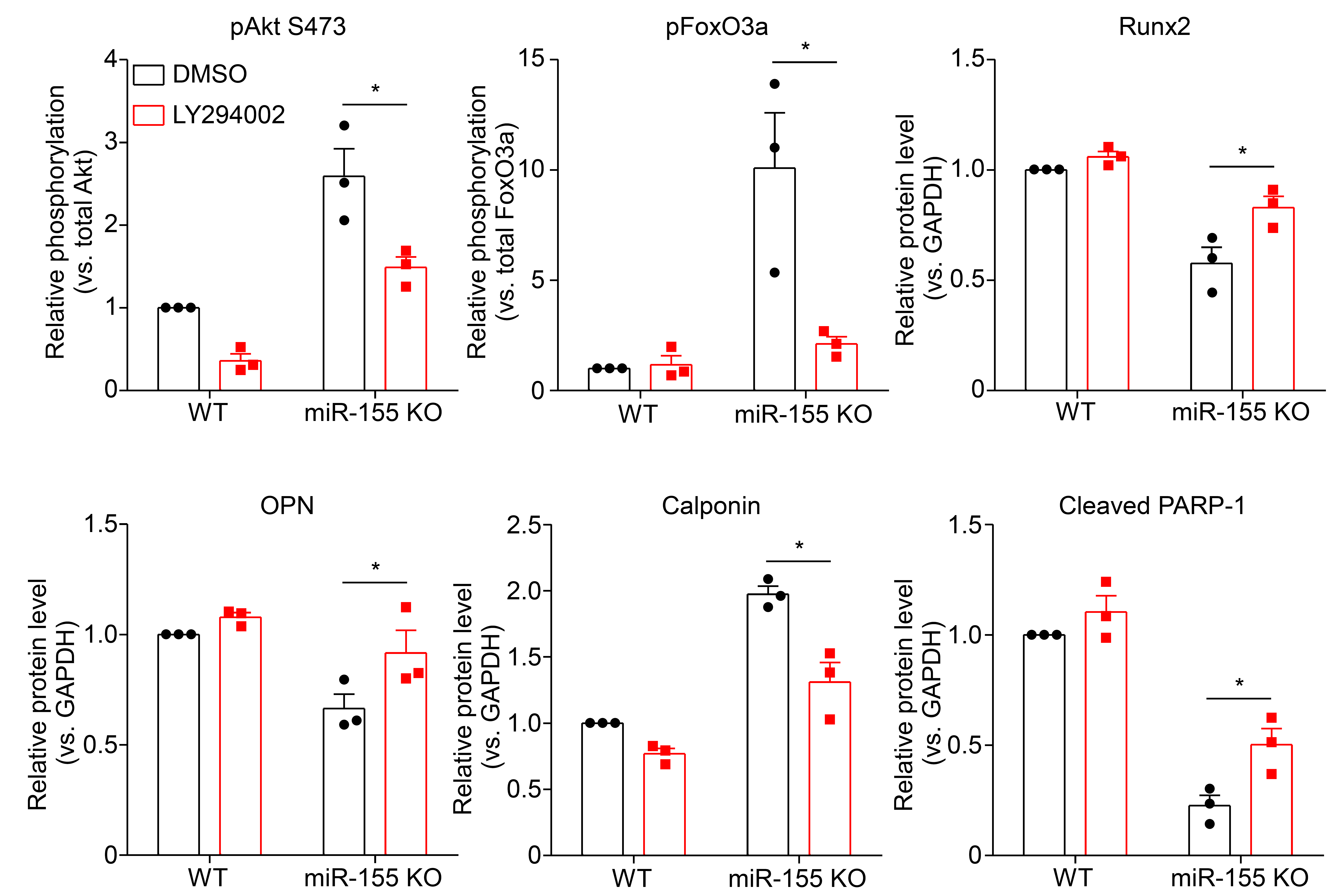


**Supplementary Figure S5.** Quantification of the data shown in Fig. 6D. Relative fold changes of protein levels or phosphorylation were determined by densitometry and normalized to GAPDH or its total protein levels, respectively, Data are presented as mean±SEM; n=6 each group; *p<0.05, two-tailed Student’s *t*-test.


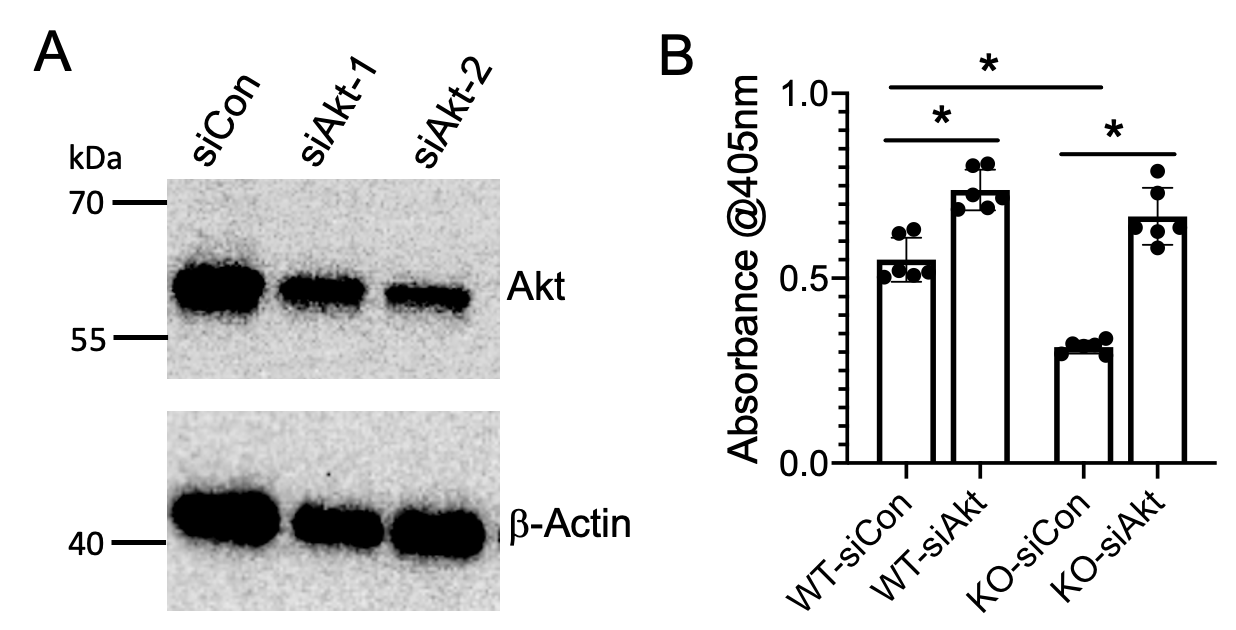

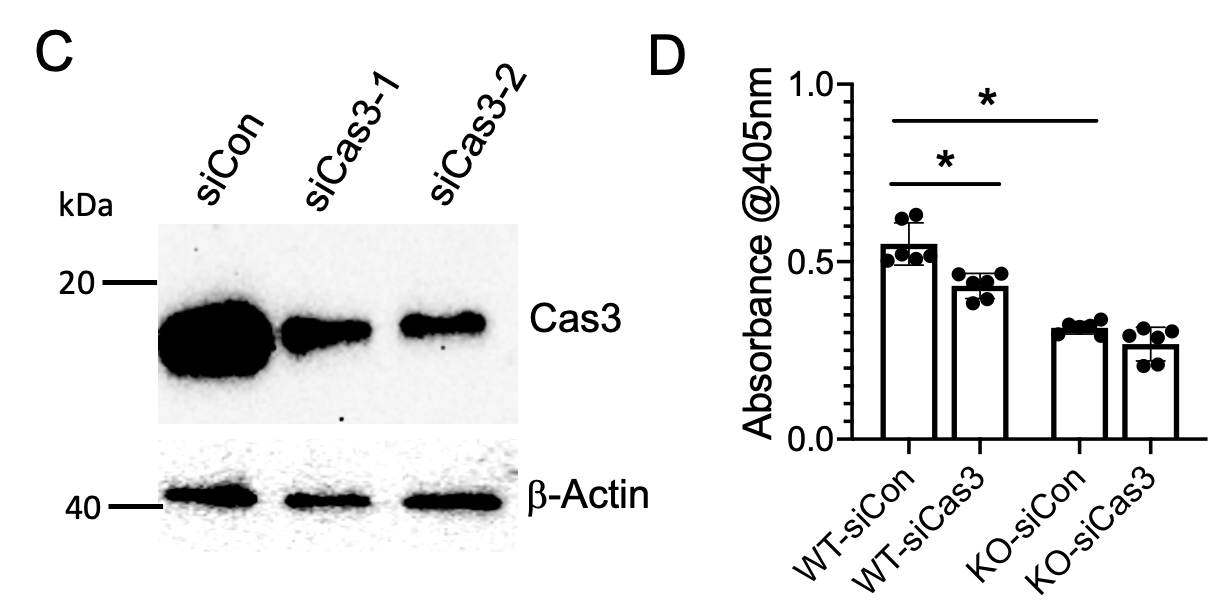


**Supplementary Figure S6. Knockdown of Akt1 and Caspase 3 altered CM-induced calcification in VSMCs.** VSMCs from WT or miR155^-/-^ mice were transfected with control, AKt1 or Caspase 3 siRNAs and treated with CM for 7 days to induce calcification. The cells were stained by Alizarin Red S and quantified by measuring Alizarin Red S concentration. **A**. Western blot shows the knockdown efficiency of Akt1 in VSMCs; **B**. Quantification of CM-induced calcification in VSMCs with Akt1 knockdown; **C**. Western blot shows the knockdown efficiency of Caspase 3 in VSMCs; **D**. Quantification of CM-induced calcification in VSMCs with Caspase 3 knockdown. N=6; *p<0.05.

**Supplementary Figure S7. A diagram depicting the mechanism by which miR155 regulates vascular calcification.** In high calcium/high phosphate condition, miR155 expression is increased in VSMCs. miR155 suppresses phosphorylation of Akt at S473 possibly through depressing Rictor/mTORC2, leading to reduced FOXO3a phosphorylation and degradation. As a result, miR155 increases FoxO3a translocation to nucleus and the transcription activities, leading to increased pro-apoptotic proteins Bim and Bax and decreased anti-apoptotic proteins Bcl-xL and Bcl-2. The tilted balance of pro-apoptotic/anti-apoptotic proteins triggers the apoptosis of VSMCs, leading to enhanced vascular calcification.
